# Association Between Standard Gait Measures and Anterior Quadriceps Muscle Thickness as Measured by Point of Care Ultrasound (POCUS) 

**DOI:** 10.24908/pocus.v9i2.17659

**Published:** 2024-11-15

**Authors:** Uyanga Ganbat, Boris Feldman, Shane Arishenkoff, Graydon S Meneilly, Kenneth M Madden

**Affiliations:** 1 Department of Medicine, Division of Geriatric Medicine, University of British Columbia Vancouver, BC CAN; 2 Department of Medicine, General Internal Medicine, University of British Columbia Vancouver, BC CAN; 3 Center of Aging SMART, University of British Columbia Vancouver, BC CAN; 4 Allan M. McGavin Chair in Geriatric Medicine, Gordon and Leslie Diamond Health Care Centre Vancouver, BC CAN

**Keywords:** Geriatric Medicine, Point-of-Care Ultrasound, Muscle Thickness, Muscle Ultrasound, Gait

## Abstract

**Background: **Gait parameters and sarcopenia both predict falls risk among older adults. Our objective was to evaluate whether fast, easy-to-obtain measures of anterior thigh muscle by point of care ultrasound (POCUS) are significantly associated with standard gait measures.** Methods:** All subjects were referred from ambulatory geriatric medicine clinics at an academic center. Quadriceps muscle thickness was measured by a portable ultrasound device. Gait variables were measured by the patient in comfortable walking shoes walking for six minutes. The primary response variables were gait variables, and the predictor variables were age, biological sex, body mass index, and muscle thickness. Univariate and multivariate regression analyses were performed.** Results: **A total of 150 participants were recruited from geriatric medicine clinics (65 women, 84 men). Muscle thickness was measured in 149 participants, and the mean (SD) was 1.91 (0.52) (median 1.82 cm, 0.96 to 3.68 cm). Univariate analysis of gait parameters with age showed a statistically significant correlation with gait speed (R^2^=0.16, P < 0.000), average stride length (R^2^=0.142, P < 0.000), and average stride velocity (R^2^=0.182, P < 0.000). Among all the gait variables, average swing time (P = 0.010) and average stance time (P = 0.010) were correlated significantly with muscle thickness. For multivariate analysis with age and gait variables, age was a significant independent variable for all gait variables that were significant in univariate analysis. **Conclusion:** POCUS showed a significant association with average swing time, average stance time, and step time variability. Although more work needs to be done, POCUS has the potential to be a rapid screening tool for gait assessment.

## Introduction

Falls are one of the major causes of mortality and morbidity, accounting for an estimated 684,000 deaths and 37.3 million hospital visits each year worldwide 1. Risk factors of falling include increasing age, white race, living alone, previous falls, cognitive impairment, and other age-related co-morbidities 2. Falls are a major public health problem for older adults due to potential fatal and nonfatal injuries resulting in decreased quality of life, increased financial expenses, and increased health services burden 3.

Walking is a basic functional marker of independence, and functional and cognitive decline related to aging leads to decline or impairment in walking 4, 5. Gait analysis has been performed to evaluate the prosthetics, diagnosis, assessment of neuropathies, and risk of falls 6. Recent advancements in wearable sensors for gait analysis have made it possible to have quantitative measures of gait parameters 7. Quantitative gait analysis measures spatial, temporal, variability, and symmetry parameters 8. Numerous studies have found an association between gait parameters, such as gait speed and stride length, and fall risk 9, 10, 11, 12, 13. For example, in community-dwelling adults, stance time and swing time were significantly different between fallers and non-fallers 14. Step time variability was associated with the risk of multiple falls in a population-based study 15. Temporal gait parameters are more sensitive to identifying future fall risk than spatial parameters 16. Among all gait parameters, slow gait speed is the most common gait analysis parameter that can be easily measured without any specific instrument. Slow gait speed was associated with falls 14, 16, and was one of the diagnostic criteria for sarcopenia 17. 

Sarcopenia is a condition where muscle mass and strength decrease. Sarcopenia further elevates the risk of falls and fractures, physical disability, impaired quality of life, and increased use of medical services 17. The standard tests for sarcopenia are magnetic resonance tomography (MRI), computed tomography (CT), dual-energy absorptiometry (DXA), and bioelectric impedance analysis. Muscle mass is one of the criteria used for sarcopenia diagnosis 17.However, when these imaging tests are only available at certain diagnostic centers, ultrasound can be a useful bedside tool for diagnosing muscle quality and quantity 17, 18, 19, 20. Skeletal muscle mass, including anterior thigh muscles, has been used to evaluate sarcopenia 21, 22, 23. Numerous papers have recorded the use of point of care ultrasound (POCUS) to measure muscle mass and thickness 24, 25, 26. 

Quantitative gait measuring requires a specific device (examples include GAITRite by CIR Systems^27^ and Kinesis Gait™ by Kinesis Health Technologies Ltd^8^ and a space for participants to walk. Since this is not possible in clinical settings and emergency situations, we proposed using the association between quadriceps muscle thickness measured by POCUS and abnormalities detected by gait analysis, such as gait speed, stance, swing time, and single and double support time, to predict falls. 

## Methods

### Subjects

Our inclusion criteria were older adults (65 years and older) who visited ambulatory geriatric medicine clinics at an academic center; they were recruited as part of an ongoing study of sarcopenia 28. We excluded patients undergoing hemodialysis, patients using chronic oral corticosteroids, and patients with hemiparesis. Furthermore, participants with pitting edema, severe hydration, and myositis were excluded. Systemic connective tissue disorders, systemic atrophies affecting the central nervous system (CNS), and CNS demyelinating disease patients were also excluded from the study. The Human Subjects Committee of the University of British Columbia approved the study protocol. All participants of the study consented to participate in writing. 

### Point of Care Ultrasound (POCUS)

Quadriceps muscle thickness was measured supine while the patients extended their knees comfortably. This position was chosen because it could be easily measured in the acute care environment, and even when a patient was too ill for formal gait assessment. 

B-mode ultrasound imaging measurements were taken by a handheld ultrasound device called Vscan with Dual probe (GE Healthcare, IL). A single operator (BF) performed all the measurements, including the femoral quadriceps cross-sectional images, as per current standards 18. 

### Gait assessment

Gait variables were measured using Kinesis Gait™ by Kinesis Health Technologies Ltd 8. A Kinesis Gait™ medical device with wireless body-worn inertial sensors was used for gait and mobility parameter assessment. The package consisted of a tablet, sensors, and accessories. The patient in comfortable walking shoes walked six meters on the floor at their usual pace. After completing the walk, the inertial sensors attached to the shins (mid-point on the anterior shank), sent data to the tablet. The available gait assessment categories were summary, temporal gait, spatial gait, gait variability, gait symmetry, and bilateral gait variables. All participants were able to perform gait assessments; however, due to the technical issues in the gait equipment, some gait data is missing. 

### Statistical methods

The primary response variables of our study were gait variables (see Table 1). Our main explanatory variables were age, biological sex, body mass index (BMI), and muscle thickness. Univariate and multivariate regression analyses were performed. Density plots were examined before statistical analysis for skewing, and skewed variables were logarithmically transformed (base 10) before both the univariate and multivariable analyses. The R software version 4.1.3 was used for statistical analysis with a significance level of P < 0.05^29^ . All data analysis was done in a blinded version and mean ± standard deviation (SD) and mean ± standard error was used to express the results. Assuming an alpha error probability of 0.05 and 90 percent power, we needed to recruit at least 109 subjects to detect a moderate effect size (moderate association) of 0.3.

## Results

A total of 149 participants were recruited from geriatric medicine clinics (65 women, 84 men), although one woman dropped out of the study before completing all measures (Table 1). Data from 64 women and 84 men were analyzed (Table 1). The mean (Standard Deviation (SD)) age of participants was 80.03 (6.03) (median age 80 years, 65 to 94 years). Muscle thickness was measured in 149 participants, and the mean (SD) was 1.91 (0.52) (median 1.82 cm, 0.96 to 3.68 cm). The mean BMI was 26.48, with an SD of 4.91. The mean (SD) average swing and stance time were both 0.51 s (0.11) (median 0.49 s, 0.34 s to 1.23 s). According to the three age categories, muscle thickness decreased by approximately 0.1 cm each decade (Table 1). Gait speed declined around 0.2 m/s each decade starting at the age of 65. Average stride length and velocity decreased significantly among the three age groups (P value <0.001). 

**Table 1:** Subject characteristics.

	**N**	**Mean (SD)**	**Age group (3 categories) **			
Age (years)		80.03 (6.03)	**65-74**	**75-84**	**85-95**	**P value**
Total participants (females)	148 (65)		17 (8)	88 (42)	43 (15)	
BMI (kg/m2)	149	26.48 (4.91)	25.86 (4.59)	26.92 (5.09)	25.85 (4.68)	0.43
Muscle thickness (cm)	149	1.91 (0.52)	2.07 (0.52)	1.92 (0.56)	1.82 (0.41)	0.216
Gait speed (m/s)	141	0.89 (0.3)	1.13 (0.29)	0.93 (0.27)	0.74 (0.27)	<0.001
Speed score percent (%)	137	60.76 (28.92)	39.9 (26.2)	57 (28.5)	77.6 (22.1)	<0.001
Variability score percent (%)	138	39.04 (16.44)	33.7 (13.8)	35.8 (14.4)	48.2 (18.3)	<0.001
Symmetry score percent (%)	138	57.04 (17.75)	50.3 (16.9)	54.9 (17.5)	64.4 (16.7)	<0.01
Recording time (s)	138	8.74 (2.45)	7.61 (1.62)	8.66 (1.93)	9.42 (3.39)	<0.05
Distance travelled (m)	137	6.62 (1.45)	7.01 (1.14)	6.79 (1.15)	6.1 (1.93)	<0.05
Average stride velocity (cm/s)	137	89.35 (29.15)	113 (28.6)	93 (26.5)	71.5 (25)	<0.001
Average stride length (cm)	137	102.4 (26.82)	119 (23)	107 (24.9)	86 (24.6)	<0.001
Number gait cycles	138	6.58 (1.46)	6 (1.22)	6.49 (1.22)	7.03 (1.88)	<0.05
Number steps	138	13.63 (2.82)	12.5 (2.29)	13.4 (2.31)	14.6 (3.67)	<0.05
Cadence steps per min (step/min)	138	106.6 (13.02)	113 (13.5)	105 (12.6)	107 (13.2)	0.087
Walk time (s)	138	7.86 (2.35)	6.75 (1.66)	7.79 (1.8)	8.49 (3.31)	<0.05
Average swing time (s)	138	0.51 (0.11)	0.48 (0.04)	0.51 (0.10)	0.53 (0.13)	0.32
Average stance time (s)	138	0.51 (0.11)	0.48 (0.04)	0.51 (0.10)	0.53 (0.13)	0.32
Average stride time (s)	138	1.15 (0.17)	1.07 (0.13)	1.16 (0.16)	1.16 (0.20)	0.156
Average step time (s)	138	0.57 (0.08)	0.54 (0.07)	0.58 (0.07)	0.58 (0.10)	0.184
Average single support time (%)	137	0.44 (0.07)	0.45 (0.04)	0.44 (0.06)	0.45 (0.09)	0.572
Average double support time (%)	132	0.14 (0.07)	0.12 (0.06)	0.14 (0.06)	0.16 (0.09)	0.138
Swing time variability percent (%)	138	9.88 (6.75)	8.03 (4.43)	8.85 (5.19)	12.8 (9.27)	<0.01
Stance time variability percent (%)	138	11.86 (11.73)	9.83 (4.49)	10 (9.12)	16.6 (16.7)	<0.01
Stride time variability percent (%)	138	6.59 (6.60)	6.12 (4.04)	6.47 (7.65)	7.05 (5.01)	0.86
Step time variability percent (%)	138	11.62 (6.80)	10.9 (4.02)	10.5 (5.22)	14.3 (9.57)	<0.05

### Univariate analysis (Table 2)

For univariate analysis, age, BMI, biological sex, and muscle thickness variables were chosen along with the standard gait parameters measured by Kinesis Gait™. BMI correlated positively to muscle thickness (R^2^=0.113, P < 0.000). Among all the gait variables, average swing time (R^2^=0.041, P = 0.010) and average stance time (R^2^=0.041, P = 0.010) correlated significantly with muscle thickness. Muscle thickness was negatively associated with step time variability percent (R^2^=0.049, P = 0.005). Univariate analysis of gait parameters with age showed a statistically significant correlation with gait speed (R^2^=0.16, P < 0.000), average stride length (R^2^=0.142, P < 0.000), and average stride velocity (R^2^=0.182, P < 0.000). The average double support time and step time variability percent were significant but had low R^2^ values (0.026 and 0.032, respectively).

**Table 2:** Univariate analysis, correlations with muscle thickness and age.

	**Muscle Thickness **			**Age **		
	**R** **^2^**	**b (SE)**	**P Value**	**R** **^2^**	**B (SE)**	**P value**
Age	0.008	-1.416 (0.958)	0.141			
Gait speed (m/s)	-0.004	2.768 (4.747)	0.561	0.160	-2.005 (0.373)	0.000***
Average stride length (cm)	-0.007	0.240 (4.375)	0.956	0.142	-1.704 (0.351)	0.000***
Average stride velocity (cm/s)	-0.007	0.0005 (0.0016)	0.765	0.182	-2.082 (0.373	0.000***
Average swing time (s)	0.041	-0.045 (0.017)	0.010*	0.002	0.002 (0.002)	0.266
Average stance time (s)	0.041	-0.045 (0.017)	0.010*	0.002	0.002 (0.002)	0.266
Average single support time (%)	0.006	-0.9239 (0.6825)	0.178	-0.006	-0.000 (0.001)	0.644
Average double support time (%)	0.003	0.7686 (0.6590)	0.246	0.026	0.002 (0.001)	0.035*
Step time variability percent (%)	0.049	-3.035 (1.074)	0.005*	0.032	0.221 (0.095)	0.021*

R^2^, coefficient of determination; b, beta-coefficient; SE, standard error; BMI, body mass index; * p < 0.05; ** p < 0.01; *** p < 0.001.

### Multivariate analysis (Tables 3 and 4)

Age, BMI, biological sex, and muscle thickness variables were entered into a multivariate analysis with standard gait variables (Table 3). Variance inflation factors (VIF) were checked, and the results indicated no multicollinearity (muscle thickness 1.18, BMI 1.15, age 1.03, and gender 1.02). We analyzed three gait parameters that were statistically significant in univariate analysis (Table 2). In our models, muscle thickness was significantly associated with average swing time (P = 0.033) and average stance time (P = 0.033). Muscle thickness (P = 0.046), age (P = 0.025), and gender (P = 0.047) also had statistically significant associations with step time variability percent. For multivariate analysis with age (Table 4), age was a significant independent variable for all gait variables. 

**Table 3:** Multivariate regression analysis with muscle thickness.

**Measure **	**R** **^2^**	**Standardised b (SE)**	**P value**
**Average swing time**	0.052		0.026*
Muscle thickness		-0.190 (0.088)	0.033*
BMI		-0.085 (0.088)	0.337
Age		0.052 (0.084)	0.535
Male gender		0.030 (0.170)	0.080
**Average stance time**	0.052		0.026*
Muscle thickness		-0.190 (0.088)	0.033*
BMI		-0.085 (0.088)	0.337
Age		0.052 (0.084)	0.535
Male gender		0.030 (0.170)	0.080
**Step time variability percent **	0.087		0.003*
Muscle thickness		-0.175 (0.087)	0.046*
BMI		-0.048 (0.087)	0.580
Age		0.187 (0.083)	0.025*
Male gender		-0.335 (0.167)	0.047*

R2, coefficient of determination; **b**, beta-coefficient; SE, standard error; BMI, body mass index, * P < 0.05.

**Table 4:** Multivariate analysis with age

**Measure **	**R** **^2^**	**Standardised b (SE)**	**P value**
**Gait speed**	0.154		0.000***
Age		-1.989 (0.377)	0.000***
Male Gender		-0.132 (4.565)	0.977
BMI		0.432 (0.459)	0.348
**Average Stride length (cm)**	0.133		0.000***
Age		-1.697 (0.355)	0.000***
Male Gender		0.482 (4.355)	0.912
BMI		0.317 (0.432)	0.464
**Average Stride velocity (cm/s)**	0.172		0.000***
Age		-2.087 (0.377)	0.000***
Male Gender		1.724 (4.625)	0.710
BMI		0.255 (0.458)	0.579
**Average double support time (%)**	0.144		0.000***
Age		0.002 (0.001)	0.017*
Male Gender		-0.007 (0.011)	0.532
BMI		0.005 (0.001)	0.000***
**Step time variability percent (%)**	0.066		0.007**
Age		0.235 (0.093)	0.013*
Male Gender		-2.457 (1.143)	0.034*
BMI		-0.152 (0.114)	0.182

R2, coefficient of determination; b, beta-coefficient; SE, standard error; BMI, body mass index; * p < 0.05; ** p < 0.01; *** p < 0.001.

## Discussion

Our study found that most gait parameters significantly change by different age groups. The average stance time, swing time, and step time variability percent had a statistically significant negative correlation with muscle thickness in healthy older adults when adjusted for age, BMI, and gender. Older adults with greater muscle thickness had lower swing and stance times. Age was a significant explanatory variable for some gait parameters adjusting for BMI and gender. 

### Previous research on relevant gait parameters 

Gait speed is the most common gait variable studied in correlation with muscle thickness and strength among many other gait parameters^30^, ^31^, ^32^ and it is included in sarcopenia and frailty diagnostic criteria as a physical performance indicator. The cut-off points for most of the studies were 1 m/s in a review article by Patrizio et al. 33, and the European Working Group on Sarcopenia in Older People 2 (EWGSOP2) recommended £0.8 m/s for severe sarcopenia 17. The mean gait speed in our study was 0.89 m/s, which is above the cut-off point by EWGSOP2. Other than the 0.74 m/s in the 85-95 age group, the lower age groups were above 0.8 m/s. 

The mean gait speed in our study by age group and gender was low (data not shown) compared to the normative values in a review study by Bohannan and Andrews 34. A correlation was found between slow gait speed and lower muscle thickness 32, 35, and our study findings match these findings in the literature. Another study by Abe et al. also showed a positive association between upper limb muscle thickness (specifically forearm-radius and forearm-ulna) and walking speed 31. This could be explained by the fact that their study participants were population-based or convenience-sampled, while our study participants were likely frailer because they were recruited from a geriatric ambulatory clinic. On top of that, gait speed, average gait speed, stride length, and cadence had lower values, and swing time and double support time were higher in our study when compared to a review study by Herssens et al. 4. 

Appendicular skeletal muscle mass in older women was associated negatively with swing phase, positively with stance phase and double support phase. In other words, higher lean mass was associated with shorter swing, longer stance and double support phases 36. In the study by Brach et al. muscle thickness was not measured, however, stance time variability was significantly associated with grip strength 37, which was correlated to muscle thickness in our pilot study 38. 

As for the step time variability, a study of CT measurement of quadriceps femoris quality among 22 institutionalized older adults found it negatively associated with muscle quality 39. Muscle function, measured by five times sit-to-stand tests among 430 older Korean women, showed a negative significant correlation with step time variability 40. 

Previous work by Lord et al. showed a significant association between quadriceps strength and gait velocity, cadence, stride length, stance duration, and composite gait score 13. The sarcopenia group had significantly shorter step and stride lengths, along with a statistically significant difference in the length of the right gait line and left single support line compared to the non-sarcopenia group 41. 

### Limitations

Our study was cross-sectional in nature, and our findings cannot illustrate causation with respect to the association between muscle mass as measured on ultrasound and the various gait parameters. In addition, participants were recruited from geriatric ambulatory clinics, which means they were a frail population with multiple co-morbidities. This limits generalizability to the broader population of older adults. We were also limited because we had only one operator doing all ultrasound and gait tests. However, studies were published on the reliability of muscle thickness measures by ultrasound. Intra- and interrater reliability was great, with the intraclass correlation coefficient being around 0.95 and the interclass correlation coefficient being 0.873 for three operators for muscle ultrasound assessment 42. Another study also confirmed the results of the first study, which had a high reliability of over 0.90 for intraclass and 0.81 for interclass correlation coefficient 43. 

### Clinical significance

POCUS has been shown as a reliable method for measuring muscle. It was comparable to bioelectric impedance analysis (BIA) among patients with chronic kidney disease^21^ and CT among intensive care unit patients 44.

Fallers had significantly lower quadriceps muscle thickness measured by ultrasound compared to non-fallers in the emergency department^45^ and in community-dwelling older adults 46. Since many factors must be accounted for to predict falls in older adults 2, one way to screen for future falls risk could be using POCUS to measure quadriceps muscle thickness in ambulatory settings. Unlike other ultrasound examinations, learning to measure muscle thickness using POCUS requires relatively little training for proficiency 47, 48. 

## Conclusion

Many gait parameters change significantly as age advances. Muscle thickness measured by POCUS was significantly correlated with average swing time, average stance time, and step time variability adjusting for age, BMI, and gender. Older adults with greater muscle thickness had lower swing and stance times. Age was a significant explanatory variable for some gait parameters adjusting for BMI and gender**.**

## Informed Consent Statement 

The Human Subjects Committee of the University of British Columbia approved the study protocol. All participants of the study consented to participate in writing. 

## Declaration of Conflict of Interest

None.

## Disclosure of Funding Statement

This study is funded by Vancouver Coastal Health Research Institute, Grant number F20-00139. 

## Authors' contributions 

UG analyzed the data and drafted the manuscript. BF collected the data. GM, SA, and KM conceptualized and designed the study methods. KM contributed to the manuscript writing and editing. All authors read and approved the manuscript. 

## References

[R254387432280222] Falls. https://www.who.int/news-room/fact-sheets/detail/falls.

[R254387432280215] Fuller G F (2000). Falls in the Elderly. Am Fam Physician.

[R254387432280230] Peel N M (2011). Epidemiology of Falls in Older Age. Can J Aging Rev Can Vieil.

[R254387432280224] Herssens N, Verbecque E, Hallemans A, Vereeck L, Van Rompaey V, Saeys W (2018). Do spatiotemporal parameters and gait variability differ across the lifespan of healthy adults? A systematic review. Gait Posture.

[R254387432280251] Sebastião E, De Melo Coelho F G, Nascimento Cmc, De Andrade L P, Pereira J R, Gobbi S (2017). The Walking Ability in Healthy Older Adults: The Role of Aging and Physical Activity and Its Interface with Agility, Balance, Cognition, and Risk of Falls. In: Barbieri FA, Vitório R, editors. Locomotion and Posture in Older Adults: The Role of Aging and Movement Disorders.

[R254387432280252] Chen S, Lach J, Lo B, Yang G Z (2016). Toward Pervasive Gait Analysis With Wearable Sensors: A Systematic Review. http://spiral.imperial.ac.uk/handle/10044/1/51564.

[R254387432280213] Prasanth H, Caban M, Keller U, Courtine G, Ijspeert A, Vallery H (2021). Wearable Sensor-Based Real-Time Gait Detection: A Systematic Review. Sensors.

[R254387432280229] Kinesis Gait assessment technology: portable low-cost, clinical-grade gait analysis. https://www.kinesis.ie/kinesis-gait/.

[R254387432280259] Gillain S, Boutaayamou M, Schwartz C, Dardenne N, Bruyère O, Brüls O (2019). Gait symmetry in the dual task condition as a predictor of future falls among independent older adults: a 2-year longitudinal study. Aging Clin Exp Res.

[R254387432280248] Moon Y, Wajda D A, Motl R W, Sosnoff J J (2015). Stride-Time Variability and Fall Risk in Persons with Multiple Sclerosis. Mult Scler Int.

[R254387432280253] Ko S U, Jerome G J, Simonsick E M, Studenski S, Ferrucci L (2018). Differential Gait Patterns by History of Falls and Knee Pain Status in Healthy Older Adults: Results From the Baltimore Longitudinal Study of Aging. J Aging Phys Act.

[R254387432280227] Ko S U, Gunter K B, Costello M, Aum H, Macdonald S, White K N (2007). Stride width discriminates gait of side-fallers compared to other-directed fallers during overground walking. J Aging Health.

[R254387432280240] Lord S R, Lloyd D G, Li S K (1996). Sensori-motor function, gait patterns and falls in community-dwelling women. Age Ageing.

[R254387432280245] Marques N R, Spinoso D H, Cardoso B C, Moreno V C, Kuroda M H, Navega M T (2018). Is it possible to predict falls in older adults using gait kinematics?. Clin Biomech Bristol Avon.

[R254387432280220] Callisaya M L, Blizzard L, Schmidt M D, Martin K L, Mcginley J L, Sanders L M (2011). Gait, gait variability and the risk of multiple incident falls in older people: a population-based study. Age Ageing.

[R254387432280228] Mortaza N, Osman Abu, Mehdikhani N A (2014). Are the spatio-temporal parameters of gait capable of distinguishing a faller from a non-faller elderly?. Eur J Phys Rehabil Med.

[R254387432280235] Cruz-Jentoft A J, Bahat G, Bauer J, Boirie Y, Bruyère O, Cederholm T (2019). Sarcopenia: revised European consensus on definition and diagnosis. Age Ageing.

[R254387432280216] Perkisas S, Baudry S, Bauer J, Beckwée D, Cock A M De, Hobbelen H (2018). Application of ultrasound for muscle assessment in sarcopenia: towards standardized measurements. Eur Geriatr Med.

[R254387432280244] Özçakar L, Ata A M, Kaymak B, Kara M, Kumbhare D (2018). Ultrasound imaging for sarcopenia, spasticity and painful muscle syndromes. Curr Opin Support Palliat Care.

[R254387432280232] Albano D, Messina C, Vitale J, Sconfienza L M (2020). Imaging of sarcopenia: old evidence and new insights. Eur Radiol.

[R254387432280218] Katari Y, Srinivasan R, Hiremathada Arvind P (2018). Point-of-Care Ultrasound to Evaluate Thickness of Rectus Femoris, Vastus Intermedius Muscle, and Fat as an Indicator of Muscle and Fat Wasting in Critically Ill Patients in a Multidisciplinary Intensive Care Unit. Indian J Crit Care Med Peer-Rev Off Publ Indian Soc Crit Care Med.

[R254387432280256] Nakanishi N, Tsutsumi R, Okayama Y, Takashima T, Ueno Y, Itagaki T (2019). Monitoring of muscle mass in critically ill patients: comparison of ultrasound and two bioelectrical impedance analysis devices. J Intensive Care.

[R254387432280247] Toledo D O, Silva Dc De L E, Santos Dmd, Freitas B J, De, Dib R, Cordioli R L (2017). Bedside ultrasound is a practical measurement tool for assessing muscle mass. Rev Bras Ter Intensiva.

[R254387432280239] Gaitrite (2024). GAITRite | World Leader in Temporospatial Gait Analysis.

[R254387432280249] Madden K M, Feldman B, Arishenkoff S, Meneilly G S (2021). A rapid point-of-care ultrasound marker for muscle mass and muscle strength in older adults. Age Ageing.

[R254387432280242] (2022). R. The R Project for Statistical Computing.

[R254387432280223] Rao N S, Chandra A, Saran S, Lohiya A (2022). Ultrasound for thigh muscle thickness is a valuable tool in the diagnosis of sarcopenia in Indian patients with predialysis chronic kidney disease. Osteoporos Sarcopenia.

[R254387432280214] Tada M, Yamada Y, Mandai K, Hidaka N (2021). Screening for sarcopenia and obesity by measuring thigh muscle and fat thickness by ultrasound in patients with rheumatoid arthritis. Osteoporos Sarcopenia.

[R254387432280231] Hida T, Ando K, Kobayashi K, Ito K, Tsushima M, Kobayakawa T (2018). Editors’ Choice Ultrasound measurement of thigh muscle thickness for assessment of sarcopenia. Nagoya J Med Sci.

[R254387432280226] Abe T, Ogawa M, Loenneke J P, Thiebaud R S, Loftin M, Mitsukawa N (2012). Relationship between site-specific loss of thigh muscle and gait performance in women: the HIREGASAKI study. Arch Gerontol Geriatr.

[R254387432280254] Abe T, Thiebaud R S, Loenneke J P, Ogawa M, Mitsukawa N (2014). Association Between Forearm Muscle Thickness and Age-related Loss of Skeletal Muscle Mass, Handgrip and Knee Extension Strength and Walking Performance in Old Men and Women: A Pilot Study. Ultrasound Med Biol.

[R254387432280237] Tsukasaki K, Matsui Y, Arai H, Harada A, Tomida M, Takemura M (2020). Association of Muscle Strength and Gait Speed with Cross-Sectional Muscle Area Determined by Mid-Thigh Computed Tomography - A Comparison with Skeletal Muscle Mass Measured by Dual-Energy X-Ray Absorptiometry. J Frailty Aging.

[R254387432280234] Patrizio E, Calvani R, Marzetti E, Cesari M (2021). Physical Functional Assessment in Older Adults. J Frailty Aging.

[R254387432280257] Bohannon R W, Andrews A Williams (2011). Normal walking speed: a descriptive meta-analysis. Physiotherapy.

[R254387432280258] Guadagnin E C, Priario Laa, Carpes F P, Vaz M A (2019). Correlation between lower limb isometric strength and muscle structure with normal and challenged gait performance in older adults. Gait Posture.

[R254387432280255] Brach J S, Studenski S, Perera S, Vanswearingen J M, Newman A B (2008). Stance Time and Step Width Variability Have Unique Contributing Impairments in Older Persons. Gait Posture.

[R254387432280260] Madden K M, Feldman B, Arishenkoff S, Meneilly G S (2021). Point-of-care ultrasound measures of muscle and frailty measures. Eur Geriatr Med.

[R254387432280250] Martinikorena I, Martínez-Ramírez A, Gómez M, Lecumberri P, Casas-Herrero A, Cadore E L (2016). Gait Variability Related to Muscle Quality and Muscle Power Output in Frail Nonagenarian Older Adults. J Am Med Dir Assoc.

[R254387432280241] Kim B, Youm C, Park H, Lee M, Choi H (2022). Association of Muscle Mass, Muscle Strength, and Muscle Function with Gait Ability Assessed Using Inertial Measurement Unit Sensors in Older Women. Int J Environ Res Public Health.

[R254387432280243] Fan Y, Zhang B, Huang G, Zhang G, Ding Z, Li Z (2022). Body Composition and Gait Analysis.

[R254387432280217] Lin Y C, Tseng I J, Lu Y C, Yang S W, Wu C C, Lin Y N (2021). Muscle Mass and Gait Characteristics in Older Women Fallers vs. Non-Fallers. J Clin Med.

[R254387432280219] Meza-Valderrama D, Sánchez-Rodríguez D, Perkisas S, Duran X, Bastijns S, Dávalos-Yerovi V (2022). The feasibility and reliability of measuring forearm muscle thickness by ultrasound in a geriatric inpatient setting: a cross-sectional pilot study. BMC Geriatr.

[R254387432280236] Lanza M B, Rock K, Marchese V, Gray V L, Addison O (2022). Ultrasound measures of muscle thickness and subcutaneous tissue from the hip abductors: Inter- and intra-rater reliability. Musculoskelet Sci Pract.

[R254387432280225] Lambell K J, Tierney A C, Wang J C, Nanjayya V, Forsyth A, Goh G S (2021). Comparison of Ultrasound-Derived Muscle Thickness With Computed Tomography Muscle Cross-Sectional Area on Admission to the Intensive Care Unit: A Pilot Cross-Sectional Study. JPEN J Parenter Enteral Nutr.

[R254387432280221] Wongtangman T, Thatphet P, Shokoohi H, Mcfadden K, Ma I, Saud A Al (2023). Association of Sonographic Sarcopenia and Falls in Older Adults Presenting to the Emergency Department. J Clin Med.

[R254387432280238] Güner M, Boğa İ, Topuz S, Baş A Okyar, Ceylan S, Çöteli S (2023). The role of ultrasonographically measured rectus femoris muscle on falls in community-dwelling older adults: a single-center study. Eur Geriatr Med.

[R254387432280246] Olgers T J, Azizi N, Bouma H R, Maaten J C Ter (2020). Life after a point-of-care ultrasound course: setting up the right conditions! Ultrasound J.

[R254387432280233] Bell G, Wachira B, Denning G (2016). A pilot training program for point-of-care ultrasound in Kenya. Afr J Emerg Med Rev Afr Med Urgence.

